# Calcium signaling around Mitochondria Associated Membranes (MAMs)

**DOI:** 10.1186/1478-811X-9-19

**Published:** 2011-09-22

**Authors:** Simone Patergnani, Jan M Suski, Chiara Agnoletto, Angela Bononi, Massimo Bonora, Elena De Marchi, Carlotta Giorgi, Saverio Marchi, Sonia Missiroli, Federica Poletti, Alessandro Rimessi, Jerzy Duszynski, Mariusz R Wieckowski, Paolo Pinton

**Affiliations:** 1 Department of Experimental and Diagnostic Medicine, Section of General Pathology, Interdisciplinary Center for the Study of Inflammation (ICSI), Laboratory for Technologies of Advanced Therapies (LTTA), University of Ferrara, Ferrara, Italy; 2 Nencki Institute of Experimental Biology, Warsaw, Poland

## Abstract

Calcium (Ca^2+^) homeostasis is fundamental for cell metabolism, proliferation, differentiation, and cell death. Elevation in intracellular Ca^2+ ^concentration is dependent either on Ca^2+ ^influx from the extracellular space through the plasma membrane, or on Ca^2+ ^release from intracellular Ca^2+ ^stores, such as the endoplasmic/sarcoplasmic reticulum (ER/SR). Mitochondria are also major components of calcium signalling, capable of modulating both the amplitude and the spatio-temporal patterns of Ca^2+ ^signals. Recent studies revealed zones of close contact between the ER and mitochondria called MAMs (Mitochondria Associated Membranes) crucial for a correct communication between the two organelles, including the selective transmission of physiological and pathological Ca^2+ ^signals from the ER to mitochondria. In this review, we summarize the most up-to-date findings on the modulation of intracellular Ca^2+ ^release and Ca^2+ ^uptake mechanisms. We also explore the tight interplay between ER- and mitochondria-mediated Ca^2+ ^signalling, covering the structural and molecular properties of the zones of close contact between these two networks.

## Introduction

Increase of intracellular [Ca^2+^] can be elicited through two fundamental mechanisms: i) Ca^2+ ^mobilization from intracellular stores, mainly the endoplasmic reticulum (ER), or ii) entry from the extracellular milieu through the opening of plasma membrane Ca^2+ ^channels. Mitochondria are equally important in physiological Ca^2+ ^signalling through a process unraveled by a series of works demonstrating that Ca^2+ ^release from the ER results in cytosolic Ca^2+ ^increases that are paralleled by similar or even larger cycles of mitochondrial calcium uptake, and subsequent release [1].

Mitochondrial Ca^2+ ^accumulation is due to the large electrochemical gradient (mitochondrial membrane potential, ψ_mt_, usually between -150 and -180 mV). Recent studies using electron tomography techniques revealed the presence of overlapping regions between ER and mitochondria separated by a minimum distance of 10-25 nm, that allows the direct physical association of ER proteins with components of the outer mitochondrial membrane (OMM) [2,3]. These zones were identified as 'hotspots' and have pivotal roles in several cellular functions, including an highly efficient transmission of Ca^2+ ^from the ER to the adjacent mitochondrial network that stimulates oxidative metabolism and, conversely, might enable the metabolically energized mitochondria to regulate ER Ca^2+ ^homeostasis [4].

**Figure 1:**
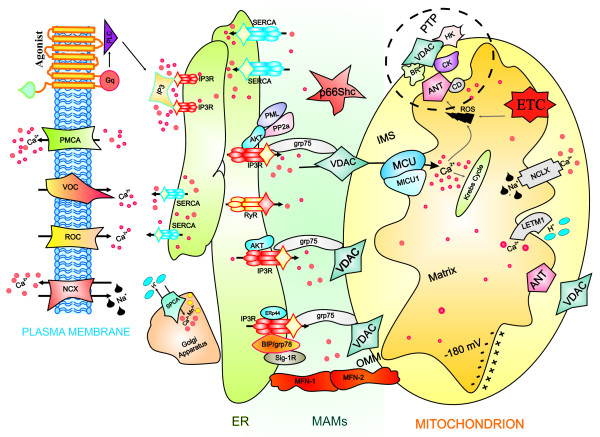
**Representation of intracellular Ca^2+ ^dynamics and MAMs proteins involved in ER-mitochondria Ca^2+ ^cross-talk**. A series of protein localized in MAMs (such as PML, AKT, grp-75, SIG-1R, Mfn-1/-2, BiP, AKT) regulate Ca^2+ ^release from the ER and an efficient mitochondrial Ca^2+ ^uptake, resulting in different functional outcomes. Cells generate Ca^2+ ^signal through two mechanism that use internal and external sources of Ca^2+^. Calcium enters into the cell through channels and pumps situated on the plasma membrane; these are gated by voltage (VOCs) or external messengers (ROCs). A series of stimuli that act on cell surface receptors triggers the activation of PLC that catalyses the hydrolysis of phosphatidylinositol 4,5-biphosphate to IP3 and DAG. The binding of IP3 to its receptor IP3R stimulates ER Ca^2+ ^release and consequently the transfer of Ca^2+ ^(red dots) from ER to mitochondria. Mitochondrial surface directly interacts with the ER through contact sites defining hotspot Ca^2+ ^signalling units. Mitochondrial Ca^2+ ^import occurs through the mitochondrial Ca^2+ ^uniporter (MCU) and the H^+^/Ca^2+ ^exchanger LETM1; conversely, NCLX, mitochondrial Na^+^/Ca^2+ ^exchanger, together with the PTP, export Ca^2+ ^from the matrix. Ca^2+ ^levels return to resting conditions through a series of channels and pumps: PMCA and NCX permit the ion extrusion into the extracellular milieu, SERCA (situated on the ER) and SPCA (on the Golgi apparatus) re-establish basal Ca^2+ ^levels in intracellular stores. Abbreviations: ANT, adenosine nucleoside transporter; ETC, electron transport chain; HK, hexokinase; CD, cyclophilin D; CK, creatine kinase; BR, benzodiazepine receptor.

In this review, we attempt to give a condensed overview of the molecular aspects of intracellular calcium homeostasis, with special emphasis on the structural and molecular aspects of the ER (main intracellular Ca^2+ ^stores), mitochondria (possessors of a series of complex systems for the influx/efflux of Ca^2+^) and MAMs (covering important roles in various cellular 'housekeeping' functions, including Ca^2+ ^signalling) (Figure 1).

## 1. Mitochondrial structure

Mitochondria, considered the "biochemical powerhouse" of the cell, are dynamic and plastic organelles, constantly subjected to remodeling, involved in a number of crucial metabolic roles, such as the tricarboxylic acid (TCA) cycle and β-oxidation of fatty acids [5]. These organelles possess two membranes that give rise to different functional regions: the inner and outer mitochondrial membranes themselves (IMM and OMM, respectively), the cytosolic side of the OMM, the intermembrane space (IMS), and the matrix. This somewhat unusual composition enables the occurrence of a wide variety of reactions, including oxidative phosphorylation. The IMM harbours complexes of the respiratory chain, ATP synthase, and enzymes involved in heme biosynthesis [2,6]. The OMM is also very rich in proteins. Small molecules (< 10 kDa) can pass freely between the cytoplasm and the IMS due to the presence of a number of porins. Conversely, the IMM is completely impermeable even to small molecules, including protons (with the exception of O_2_, CO_2_, and H_2_O). This peculiarity enables the complexes of the respiratory chain to build up a proton gradient across the IMM, required for oxidative phosphorylation. The resulting electrochemical proton gradient forms the basis of the ψ_mt _and is utilized to generate chemical energy in the form of ATP. This ψ_mt _is also one of the crucial factors responsible for Ca^2+ ^uptake. Ca^2+ ^can freely cross the OMM but in order to enter the matrix it must pass through the mitochondrial Ca^2+^uniporter (MCU), an IMM-located channel driven by a large electrochemical gradient [7-10].

## 2. ER structure

The endoplasmic reticulum (ER) is considered the largest individual intracellular organelle. It consists of a three-dimensional network of endomembranes, constituting a complex grid of microtubules and cisternae. The domains accounting for the ER are functionally and structurally distinct. Historically, ER histology describes three types of ER: smooth ER, rough ER and the nuclear envelope (NE) [11]. This division demonstrates the individual role of each particular ER type. A morphological characterization describes the NE and peripheral ER, the later being a network of tubules and sheets reaching the most remote areas of the cell. Such structural diversity of the ER, reviewed extensively by a number of authors [12-15], is related to the variety of cellular functions played by the organelle. For such a large organelle, the ER is unexpectedly plastic [16]. This plasticity is a crucial morphological characteristic of the ER and the remodeling ability seems to be correlated with the diversity of its functions. The most important activities of ER regard protein synthesis and maturation. Protein synthesis occurs in ribosome-rich rough ER, whereas their post-translational processing is carried out by an extensive group of chaperone proteins [17]. Importantly, the ER acts as a transport route enabling the delivery of a number of proteins to their destination [18]. The third function is that the ER is a dynamic reservoir of Ca^2+ ^ions, which can be activated by both electrical and chemical cell stimulation [16,19]. This feature renders this organelle an indispensable source of Ca^2+ ^in many aspects of physiological signalling.

## 3. MAM structure

The association between the ER and mitochondria was first described by Copeland and Dalton over 50 years ago in pseudobranch gland cells [20]. However, it was only in the beginning of the 1970s that mitochondria-ER contacts were visualized by a number of research groups [21,22]. Further developments in microscopy techniques provided researchers with the ability to perform detailed analyses with high resolution in 3 dimensions [23,24]. The interactions between these organelles at the contact places are so tight and strong that subcellular fractionation enabled the performance of a step through which a unique fraction, originally named as 'mitochondria-associated membranes" (MAMs fraction), can be isolated [25]. Further developments in experimental procedures enabled scientists to isolate pure MAM fractions from yeast, different organs and tissues, as well as various cell lines [25,26].

## 4. The intracellular Ca^2+^-signalling network

Intracellular Ca^2+ ^signalling is versatile and fundamental for the regulation of multiple cellular processes, including development, proliferation, secretion, gene activation and cell death. The universality of Ca^2+ ^as an intracellular messenger depends on its enormous versatility in terms of speed, amplitude, and spatio-temporal patterning. The Ca^2+^-signalling network can be divided into four functional units:

• signalling is triggered by a stimulus that generates various Ca^2+ ^-mobilizing signals;

• the latter activate the ON mechanisms that feed Ca^2+ ^into the cytoplasm;

• Ca^2+ ^functions as a messenger to stimulate numerous Ca^2+^-sensitive processes; and finally,

• the OFF mechanisms, composed of pumps and exchangers, remove Ca^2+ ^from the cytoplasm to restore the resting state [27].

Inside cells, Ca^2+ ^concentration ([Ca^2+^]) is controlled by the simultaneous interplay of multiple counteracting processes, which can be split into "ON mechanisms" and "OFF mechanisms". With these, cells maintain a rigid control over the cytosolic level of Ca^2+^. In resting conditions, cells maintain a low Ca^2+ ^concentration in the cytoplasm (around 100 nM). Ca^2+ ^influx from the extracellular space (that posses a concentration around 1-2 mM), or Ca^2+ ^release from intracellular Ca^2+ ^stores, such as the ER (with concentrations of 250-600 μM) generate the intracellular calcium signals [28].

## 5. The Ca^2+^-induced Ca^2+ ^release process

Cells generate their Ca^2+ ^signals through two fundamental mechanisms that make use of internal and external sources of calcium. Ca^2+^-mobilizing signals from internal stores are generated by stimuli that act through a variety of cell-surface receptors, such as G-protein-linked receptors and receptor tyrosine kinases. When a ligand binds its specific receptor in the plasma membrane, the occupied receptor causes GDP-GTP exchange on an associated G-protein, Gq, that activates a specific membrane-bound phospholipase C (PLC), which in turn catalyzes the production of the two second messengers, diacylclycerol (DAG) and inositol-1,4,5-trisphosphate (IP3), by hydrolysis of phosphatidylinositol 4,5-bisphosphate in the plasma membrane. IP3, a water-soluble compound, diffuses from the plasma membrane to the endoplasmic reticulum, where it binds to specific IP3 receptors (IP3Rs) and causes Ca^2+ ^channels within the ER to open. Sequestered Ca^2+ ^is thus released into the cytosol, and the cytosolic [Ca^2+^] rises sharply to about 10 μM [29].

On the plasma membrane there is a large family of Ca^2+ ^entry channels, important for the "ON reaction", which can be defined by the way in which they are activated. The most known are voltage-operated channels (VOCs) that are triggered by membrane depolarization. Other channels, called receptor-operated channels (ROCs), are sensitive to the binding of different external signals, usually transmitters. Finally, store-operated channels (SOCs), respond to the depletion of internal Ca^2+ ^stores and contribute to the spatio-temporal pattern of Ca^2+ ^waves [30].

As mentioned above, IP3Rs are the most important actors in Ca^2+ ^release from internal stores, controlled by Ca^2+ ^itself that acts either on the luminal or cytoplasmic sides of the channel.

### 5.1 *IP3Rs*

IP3Rs, the main Ca^2+^-release channels in the ER of most cell types, consist of four subunits of about 310 kDa each with a similar general structure. In mammals, three different genes encode for three different isoforms (IP3R1, -2 and -3). Structurally, the proteins have a cytoplasmic N-terminal hydrophobic region, predicted to contain six membrane-spanning helices, and a relatively short cytoplasmic C-terminus. Functionally, the N-terminal domain contains the IP3-binding domain and a "regulatory"/"coupling" domain. IP3Rs can be modulated primarily by IP3 and Ca^2+ ^itself; the latter can also regulate IP3Rs indirectly through calmodulin (CaM); other modulators include phosphorylation by Ca^2+^/CaM-dependent kinase II (CaMKII), cGMP-dependent protein kinase (PKG), protein kinase C (PKC), and cAMP-dependent protein kinase (protein kinase A, PKA). This suggests that the IP3R works as a crosstalk station between Ca^2+ ^signalling and phosphorylation [31]. As a confirmation of this, IP3R isoforms contain multiple phosphorylation consensus sites and many docking sites for protein kinases and phosphatases, and at least 15 different protein kinases are known to directly phosphorylate IP3R [32].

## 6. Mitochondria in calcium signalling

Mitochondria are important components of the "OFF" reaction since they modulate both the amplitude [33] and the spatio-temporal patterns of Ca^2+ ^signals [34,35].

That mitochondria can accumulate certain ions from the suspending medium was first observed in the early 1960s, when was discovered that isolated mitochondria from rat liver, kidney, brain and heart can accumulate large net amounts of Ca^2+ ^from the suspending medium during electron transport, up to several hundred times the initial Ca^2+ ^content [36].

In these studies, initial velocities of energy-dependent Ca^2+ ^uptake were measured by stopped-flow and dual-wavelength techniques in mitochondria isolated from hearts of rats. The first rate of Ca^2+ ^uptake shows that the initial velocity of Ca^2+ ^uptake was slow at low concentrations of Ca^2+ ^and increased sigmoidally to 10 nM Ca^2+^/s/mg protein at 300 μM Ca^2+^. Similar results were obtained by the employment of mitochondria subjected of a wide range of mitochondrial protein in the medium (0.5-10 mg/ml), when these organelles were oxidizing glutamate-malate and when acetate was replacing phosphate as a permanent anion [37].

Comparable rates of Ca^2+ ^uptake and sigmoidal plots were obtained with mitochondria from other mammalian hearts, as like guinea pigs, squirrels, pigeons, and frogs where the rate of Ca^2+ ^uptake was 0.05 nM/mg/s at 5 μM Ca^2+ ^and increased sigmoidally to 8 nM/mg/s at 200 μM Ca^2+ ^[38].

Mitochondrial Ca^2+ ^uptake plays a key role in the regulation of many cells functions, ranging from ATP production to cell death. Increases in mitochondrial calcium activates several dehydrogenases and carriers, inducing an increase in the respiratory rate, H^+ ^extrusion, and ATP production necessary for the correct energy state of the cell. However, prolonged increase in [Ca^2+^]_m _leads to the opening of the mitochondrial permeability transition pore (PTP), a critical event driving to cell death by apoptosis [39].

Although it is generally accepted that cellular energy metabolism, survival and death are controlled by mitochondrial calcium signals, the underlying molecular mechanisms have been completely elucidated yet. Several studies have identified three essential proteins mediating the processes of calcium influx and efflux.

### 6.1 Mitochondrial Calcium Uniporter (MCU)

The main transporters involved in the uptake of Ca^2+ ^into mitochondria is the MCU, characterized by a low affinity for Ca^2+^; in fact, MCU takes up Ca^2+ ^in the micromolar range and experiments in permeabilized cells report a *K_d _*of the uniporter of 10 μM [40]. In addition, a biphasic effect of calcium on the MCU has been reported: beyond a certain level, cytosolic Ca^2+ ^inactivates the uniporter, preventing further Ca^2+ ^uptake and this process might avoid an excessive accumulation of the cation in mitochondria [41].

In spite of repeated efforts by different researchers, the molecular identity of the MCU has remained elusive. Among the early candidates proposed for the MCU were the uncoupling proteins UCP2/3 [42], but experiments in different tissues of mice lacking UCP2 and UCP3 showed a normal Ca^2+ ^uptake [43]. Recently, Perocchi and colleagues [44] demonstrated that MICU1 (mitochondrial calcium uptake 1), also known as FLJ12684 or CBARA1, has a key role in regulating the classically defined uniporter. MICU1 is associated with the IMM and has two canonical EF hands that are essential for its activity and it caused a significant suppression of the [Ca^2+^]_m _signal evoked by an IP3-linked agonist. Silencing MICU1 does not impair mitochondrial respiration or membrane potential but abolishes Ca^2+ ^entry in intact and permeabilized cells, and attenuates the metabolic coupling between cytosolic Ca^2+ ^transients and activation of matrix dehydrogenases.

More recently, in 2011, two distinct laboratories have been identified a transmembrane protein (CCDC109A) that fulfilling the criteria for being the MCU [9,10]. Indeed, in planar lipid bilayers CCDC109A showed channel activity with electrophysiological properties as those previously reported for the MCU [8]. The over-expression of CCDC109A (that now is called "MCU"), increases mitochondrial Ca^2+ ^uptake and sensitizes cells to apoptotic stimuli, and the employment of short interfering RNA (siRNA) silencing of MCU strongly reduced mitochondrial Ca^2+ ^uptake. This reduction is specific for mitochondria (Ca^2+ ^cytosolic levels remain almost unaffected), does not induce impairment of the electrochemical gradient or change in mitochondrial morphology and the induction of specific mutations at the level of the putative pore-forming region reduce the mitochondrial calcium uptake and blocks the channel activity of the protein [9,10].

To conclude, MCU and MICU1 are critical for the correct mitochondrial calcium uptake: the first one can be considered the main component of the uniporter, while MICU1 as a fundamental regulator.

### 6.2 LETM1

As described above, MCU only takes up Ca^2+ ^in the micromolar range, but evidence has shown that mitochondria are able to take up Ca^2+ ^also at much lower concentrations, as recently reported by Jang and colleagues who identified a high-affinity mitochondrial Ca^2+^/H^+ ^exchanger capable of importing calcium in the nanomolar range [45]. This group conducted a genome-wide RNAi screen in *Drosophila *cells stably expressing a mitochondria-targeted ratiometric Pericam and identified the gene CG4589 (*Drosophila *homolog of the human gene LETM1, leucine zipper-EF-hand containing transmembrane protein 1) as a regulator of mitochondrial Ca^2+ ^and H^+ ^concentrations, supporting electrogenic import of Ca^2+ ^(one Ca^2+ ^in for one H^+ ^out).

However, the effective role of LETM1 as Ca^2+^/H^+ ^exchanger still remains a subject of discussion, since its activity is blocked by treatment with CGP37157 (channel inhibitor that mediates mitochondrial calcium efflux) and red/Ru360 (inhibitor of MCU). Furthermore, LETM1 is associated with K^+ ^homeostasis, and the loss of LETM1 lowers mitochondrial membrane potential, and the mitochondrial H^+^/Ca^2+ ^exchanger turned out to be non-electrogenic (one Ca^2+ ^in for two H^+ ^out) [45,46].

### 6.3 NCLX/NCKX6

A Na^+^-dependent mechanism that mediates mitochondrial Ca^2+ ^efflux has been demonstrated, but the molecular identity of this transporter has also remained elusive. In a recent study, Palty and co-workers showed that the Na^+^/Ca^2+ ^exchanger NCLX is enriched in mitochondria, where it is localized to the cristae [47]. This protein was identified as a member of the Na^+^/Ca^2+ ^exchanger situated in the ER or plasma membrane, but Palty et al., shown that in several tissues endogenous NCLX is enriched primarily in mitochondria, but not in ER and plasma membrane. The same observation is achieved overexpressing the protein in different cell lines, and the results show that expression of NCLX enhances mitochondrial Ca^2+ ^efflux; this is blocked by CGP37157 and by mutations in the catalytic site of NCLX. Besides, the role of NCLX as a mitochondrial Na^+^/Ca^2+ ^exchanger is supported by evidence that NCLX mediates Li^+^/Ca^2+ ^exchange, a functional property that, among NCX proteins, is shared exclusively with the mitochondrial exchanger [47].

## 7. Intracellular calcium extrusion mechanism

Once it activates its downstream targets, Ca^2+ ^has carried out its functions and needs to be rapidly removed from the cytosol to restore the resting levels of approximately 100 nM. For this purpose, the cell uses the combined activity of Ca^2+ ^extrusion mechanisms (such as PMCA and NCX) and mechanisms that refill the intracellular stores (like sarco-endoplasmic reticulum Ca^2+-^ATPases, SERCAs, and the secretory-pathway Ca^2+^-ATPases, SPCAs, of the Golgi apparatus).

### 7.1 PMCA

The plasma membrane calcium ATPases (PMCA) is localized on the plasma membrane and couples ATP hydrolysis to the maintenance of appropriate cytoplasmic calcium levels by removing calcium from the cytosol to the extracellular spaces. There are at least four different PMCA isoforms (PMCA1-4) and several splice variants (about 26) that are encoded by four independent genes. Some of these are ubiquitously expressed in the organism (PMCA1 and -4), while others (PMCA2 and -3) have a tissue-specific expression patterns. Structurally, PMCAs consist of 10 transmembrane domains, two major intracellular loops, and N- and C-cytoplasmic domains. The pump operates with high Ca^2+ ^affinity and low transport capacity, with a 1:1 Ca^2+^/ATP stoichiometry. Under optimal conditions, the *K_d _*of PMCA for Ca^2+ ^is about 10-30 μM in resting conditions and about 0.2-0.5 μM in activated conditions [48].

It has been demonstrated that PMCA operates as a Ca^2+^/H^+ ^exchanger and, even if the exact stoichiometry is not well defined, a recent study suggests that the Ca^2+^:H^+ ^ratio is 1:2 and that the activity of the pump is insensitive to variations of membrane potential [49].

### 7.2 NCX

NCX (Na^2+^/Ca^2+ ^exchanger) is a plasma-membrane enzyme, mainly located in excitable tissue, which carries out the efflux of one Ca^2+ ^against an influx of 3 Na^+^. NCX easily reverses its direction and brings Ca^2+ ^into the cells if the Na^+ ^concentration gradient decreases and/or the membrane potential becomes less negative. Three distinct genes are known to encode as many isoforms, namely NCX1, NCX2 and NCX3 [50]. The first one has an ubiquitous distribution, while NCX2 is expressed primarily in the brain, and NCX3 in the skeletal muscle. The NCX1 protein contains 11 putative transmembrane domains, divided into two sets of putative transmembrane domains separated by a large intracellular loop mainly responsible for the transport of the Na^+ ^and Ca^2+ ^across the membrane [29].

### 7.3 SPCA

SPCAs are the newest addition to the family of phosphorylation-type ATPases and they are responsible for supplying the lumen of the Golgi apparatus with Ca^2+^. Unlike other Ca^2+^-ATPase pumps, SPCA pumps are not electrogenic; in fact, they do not counter-transport H^+ ^to the outside since protons are essential for the correct development and functioning of the Golgi vesicle [51]. In addition to Ca^2+ ^transport, the most important property of SPCA pumps is also to transport efficiently Mn^2+ ^into the Golgi, as this is a necessary cation for enzymes present in the lumen of the Golgi compartment. SPCAs function through a reversibly cycle between an E1- and an E2-conformation. In the cytosol, the high-affinity binding site of the protein in E1-conformation binds Ca^2+ ^(or Mn^2+^), and phosphorylation by ATP creates an high-energy phosphoenzyme intermediate. This enzyme undergoes a rate-limiting transition to the lower-energy state, E2, and simultaneously Ca^2+ ^(or Mn^2+^) moves through the transmembrane pore and is released into the lumen of the Golgi apparatus. As a last step, the protein returns to the dephosphorylated state [52].

### 7.4 SERCA

SERCA is a pump identified in 1961-1962 in a skeletal muscle fraction. It is localized in the membranes of endo(sarco)plasmic reticulum and couples ATP hydrolysis to the transport of Ca^2+ ^from cytoplasm to lumen. Early studies shown that the pump counter-transported H^+ ^in exchange for two Ca^2+ ^per ATP hydrolyzed. However, it has been noticed that fewer than four H^+ ^were released to the cytosol per two Ca^2+ ^pumped, showing that the transport reaction was only partly electrogenic [53]. Like other Ca^2+^-ATPase pumps, SERCAs exist in two conformational states. The E1 on the cytosolic site, in which the enzyme has high Ca^2+ ^affinity, and the E2 state, in which the lower Ca^2+ ^affinity leads to the release of Ca^2+ ^on the opposite side. This cycle has a number of other states that occur upon binding of Ca^2+^, involving a series of structural changes in the cytoplasmic sector and in the transmembrane domain, necessary for completing the catalytic cycle. The peculiarity of SERCAs in respect to the other Ca^2+^-ATPase pumps is to have two Ca^2+ ^binding sites, enabling the existence of a Ca^2+^/ATP transport stoichiometry of 2.0 [54].

## 8. MAMs, a functional link between ER and mitochondria

As described above, mitochondria and endoplasmic reticulum networks are fundamental for the maintenance of calcium homeostasis. Recently, different studies have documented the crucial role that MAMs play in intracellular Ca^2+ ^signalling. The physical proximity of the ER to mitochondria enables a direct, selective transmission of physiological and pathological Ca^2+ ^signals [1], an aspect highly variable between cell types. In fact, mitochondria are not always morphologically continuous, functionally homogenous and associated to ER. At demonstration of this, different works revealed the existence of a largely interconnected mitochondria network akin to ER in HeLa cells, COS-7 cells, cardiac myocytes and rat hepatocytes [24,55]. Contrary, it has been also reported that mitochondria can exist as two distinct populations, one in perinuclear position and the other one in cell periphery, with different biochemical and respiratory properties [56]. Or, again, mitochondria within individual cells are morphologically heterogeneous and appear as distinct entities [57]. These different aspects could carry out diverse aspect of mitochondrial functions, in particular Ca^2+ ^sequestration, fundamental for the regulation of mitochondrial metabolism and regulation of apoptosis [39]. Lately, it has been demonstrated that the juxtaposition between ER and mitochondria is also regulated by cellular status. In fact, a condition of starvation (an autophagic trigger) leads to PKA activation, which in turn phosphorylates the pro-fission dynamin-related protein 1 (DRP1) with consequent mitochondria elongation in a network of highly interconnected organelles. This mitochondrial elongation protects cells from death and is required to sustain ATP levels and viability [58].

The MAMs, these ER-contiguous membranes, contain multiple phospholipid- and glycosphingolipid-synthesizing enzymes, including long-chain fatty acid-CoA ligase type 4 (FACL4) and phosphatidylserine synthase-1 (PSS-1), enzymes required for cholesterol and ceramide biosynthesis, enzymes involved in glucose metabolism and support direct transfer of lipids between the ER and mitochondria [1,59]. MAMs also constitute a calcium signaling hub regulating ER chaperone-assisted folding of newly synthesized proteins, the mitochondria-localized dehydrogenases activity, and the activation of Ca^2+^-dependent enzymes that execute cell death programs [60]. The importance of MAMs began to emerge when it was found that, after cell stimulation, mitochondria were able to uptake Ca^2+ ^directly from IP3Rs [8,61]. It was thus possible to identify specialized signalling microdomains, selectively enriched in critical Ca^2+ ^signalling elements, labelled as "hotspots zones", where Ca^2+ ^is transferred from the ER into mitochondria (Table 1). The interactions between the two organelles are modulated by mitochondria-shaping proteins and chaperone proteins. MFN-1 and -2 (mitofusin-1 and -2) belong to the first group and stabilize the interaction between adjacent mitochondria, regulate ER morphology and calcium homeostasis, and directly tether ER to mitochondria, thus facilitating efficient Ca^2+ ^uptake by mitochondria [23]. The hsp70 homologous cytosolic chaperone grp75 (glucose-regulated protein 75) tethers the N-terminal domain of the type-1 IP3Rs to the isoform 1 of VDAC, generating a molecular bridge that enhances the Ca^2+ ^accumulation in mitochondria [62].

**Table 1:** MAMs proteins involved in ER-mitochondria Ca^2^^+ ^cross-talk and relative functions

ACRONYM	FULL NAME	FUNCTION	LITERATURE
**AKT/PKB**	protein kinase B	Ca^2+ ^signaling, apoptosis	[70]

**PP2a**	Protein phosphatase 2	Ca^2+ ^signaling, apoptosis	[70]

**ANT**	adenine nucleotide translocase	Part of mitochondrial contact sites and/or PTP	[71]

**BAP31**	B-cell receptor-associated protein 31	Ca^2+ ^signaling, apoptosis	[72]

**Carleticulin**	carleticulin	Ca^2+ ^handling	[73]

**ERp44**	endoplasmic reticulum resident protein 44	Ca^2+ ^handling	[74]

**grp75**	glucose-regulated protein 75	Ca^2+ ^handling	[62]

**grp78**	78 kDa glucose-regulated protein	Ca^2+ ^handling	[75]

**IP3R**	inositol 1,4,5-triphosphate receptor	Ca^2+ ^handling	[62,76]

**p66Shc**	the 66 kDa isoform of ShcA protein	ROS production and signal transduction	[69]

**PACS-2**	phosphofurin acidic cluster sorting protein 2	Protein sorting, Ca^2+ ^handling	[77]

**PEMT2**	phosphatidylethanolamine N-methyltransferase 2	Lipids synthesis and trafficking	[78]

**PML**	promyelocytic leukemia protein	Ca^2+ ^handling	[70]

**PSS-1 and -2**	phosphatidylserine synthase 1 and 2	Lipids synthesis and trafficking	[79]

**Serca2b**	sarcoplasmic reticulum calcium ATPase 2b	Ca^2+ ^handling	[80]

**Sig-1R**	sigma-1 receptor	Ca^2+ ^handling	[63]

**tSERCA1**	truncated sarco(endo)plasmic reticulum Ca^2+ ^ATPase	Ca^2+ ^leak from ER	[81]

**VDAC**	voltage-dependent anion channel	Chanel, Ca^2+ ^handling	[76]

**sphingolipid-specific Glc-T**	sphingolipid-specific glycosyltransferases	Lipids synthesis and trafficking	[82]

**S100B**	S100 calcium binding protein B	Lipids synthesis and trafficking, MAM stabilization	[83,84]

**apoE, apoB and apoC**	apolipoproteins	lipid trafficking	[79,85]

**p58**	58 kDa protein	lipid trafficking	[79]

**DGAT**	diacylglycerol acyltransferase	Lipids synthesis	[79]

**Ankyrin-B**	ankyrin-B	Ca^2+ ^handling	[86]

**CNX**	calnexin	Ca^2+ ^handling	[80,87]

**Bcl-2**	B-cell lymphoma 2 protein	Ca^2+ ^homeostasis, apoptosis	[88]

**Bcl-XL**	B-cell lymphoma-extra large protein	Ca^2+ ^homeostasis, apoptosis	[88]

**MFN 1 and 2**	Mitofuzin 1 and 2	MAM stabilization	[23]

**TIM & TOM complexes**	Transporter Inner Membrane complex & Transporter Outer Membrane complex	Protein transport	[89,90]

**vMIA/HCMV**	The human cytomegalovirus	ERmitochondrial cross-talk	[91]

**p7 and NS5B protein**	proteins of hepatitis C virus	ERmitochondrial cross-talk	[92]

**ACAT**	acyl- CoA:cholesterol acyltransferase	Lipids synthesis	[79]

**Ero1α**	ER oxidase 1 alpha	Ca^2+ ^signaling	[93]

Recently identified, the Sigma-1 ER receptor (Sig-1R) selectively resides at the MAMs, forms a Ca^2+^-sensitive chaperone complex with BiP/GRP78 (78-kDa glucose-regulated protein GRP78, also referred to as the immunoglobulin binding protein BiP) and associates with isoform 3 of IP3R. Upon activation of IP3Rs, which causes the decrease of Ca^2+ ^concentration at the MAM, redistribution of Sig-1Rs occurs, from MAMs to the periphery of the ER: here Sig-1Rs dissociates from BiP/GRP78 and the chaperone activity of free Sig-1Rs attenuates the aggregation of IP3R3 [63].

Obviously, other proteins that are required to modulate calcium mobilization upon cellular stimulation are directed to MAMs. An example is the anti-apoptotic protein AKT/PKB that, in response to survival signals, is recruited to MAMs in order to inactivate IP3R3, significantly reducing ER-Ca^2+ ^release activity with a diminished cellular sensitivity to apoptotic stimuli [64]. In turn, this event determines the PML (promyelocytic leukemia protein)-mediated recruitment of phosphatase PP2a (protein phosphatase 2a) at the MAMs to switch off the kinase. Also cytochrome *c*, which is released from mitochondria upon activation of apoptotic pathways, can bind IP3Rs at the MAMs, further activating the Ca^2+ ^flux and enhancing apoptotic signaling [65]. Recently, two interesting proteins with a marked regulatory effect on cell survival through changes in Ca^2+ ^have been identified in the zones of mitochondria-ER association.

p66Shc (a 66-kDa isoform of the growth factor adapter Shc) is a cytosolic adaptor protein, profoundly involved in the cellular response to oxidative stress. This protein has also been found in the MAM fraction. Its direct relation to mitochondrial ROS production has been repeatedly documented, also by our groups [66-68]. We found that the level of p66Shc in the MAM fraction is age-dependent and corresponds well to the mitochondrial ROS production which is found to increase with age [69].

**Figure 2:**
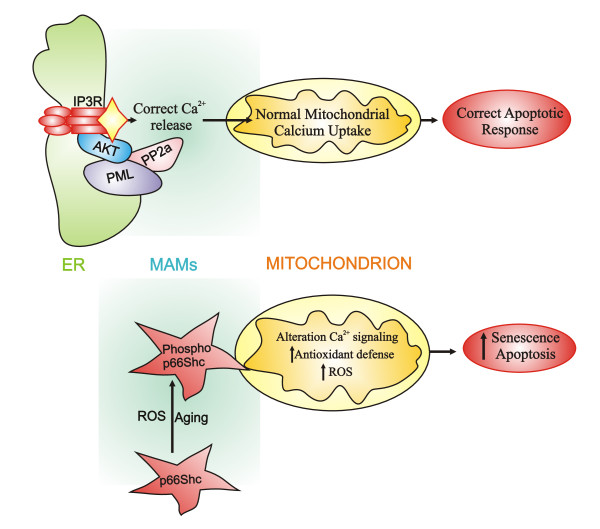
**PML and p66Shc regulates cell span at the MAMs level**. The tumor suppressor PML in resting conditions resides in a specific multi-protein complex with IP3R, PP2a and AKT, essential for a normal Ca^2+ ^flux from ER to mitochondria and, consequently, for correct apoptosis levels (upper panel). Aging and ROS determine phosphorylation and accumulation of p66Shc in the MAMs fraction. The presence of phospho-p66Shc at the mitochondrial level determines alterations in mitochondrial homeostasis, including Ca^2+ ^signalling, and ultimately increases apoptotic and senescence responses (lower panel).

PML is another protein recently identified in the MAM fraction. There, it is believed to regulate the ER machinery responsible for Ca^2+ ^release. The lack of PML results in a decreased Ca^2+ ^release from the ER and a subsequent lower Ca^2+ ^influx into mitochondria. Detailed studies on the role of PML protein in the MAM fraction contributed to the formation of the hypothesis that this protein regulates cell survival through the ER-cytosol/mitochondria calcium signalling [70] (Figure 2).

## Conclusion

In this review, we have sketched out the main features of the intracellular Ca^2+^-signalling toolkit and the elaborate relationship between ER and mitochondria. It has been demonstrated that the physical interactions between ER and mitochondria, known as MAMs, are essential for functions of the two organelles, and that these also enable an highly efficient transmission of Ca^2+ ^from the ER to mitochondria. In this regard, it becomes evident that the ER-mitochondria interface points deeply affect intracellular Ca^2+ ^signalling and are fundamental for different functional outcomes, such as cell metabolism or induction of cell death.

## List of abbreviations

ψ_mt_: mitochondrial membrane potential; AKT/PKB: Protein kinase B; BiP/GRP78: Binding immunoglobulin Protein/78-kDa glucose-regulated protein; Ca^2+^: calcium ions; [Ca^2+^]: Ca^2+ ^concentration; [Ca^2+^]_c_: cytosolic Ca^2+ ^concentration; [Ca^2+^]_m_: mitochondrial Ca^2+ ^concentration; CaM: calmodulin; CaMKII: calmodulin-dependent protein kinase II; DAG: diacylclycerol; DRP1: dynamin-related protein 1; ER: endoplasmic reticulum; FACL4: long-chain fatty acid-CoA ligase type 4; grp75: glucose-regulated protein 75; IMM: inner mitochondrial membrane; IMS: intermembrane space; IP3: inositol 1,4,5-trisphosphate; IP3R: inositol 1,4,5-trisphosphate receptor; Letm1: leucine zipper-EF-hand containing transmembrane protein 1; MAMs: mitochondria-associated membranes; MCU: mitochondrial Ca^2+ ^uniporter; MICU1: mitochondrial calcium uptake 1; MFN-1/-2: mitofusin-1: -2; NCX: Na^2+^/Ca^2+ ^exchanger; NE: nuclear envelope; OMM: outer mitochondrial membrane; p66Shc: 66-kDa isoform of the growth factor adapter shc; PKA: protein kinase A; PKC: protein kinase C; PKG: cGMP-dependent protein kinase; PLC: phospholipase C; PMCA: plasma membrane Ca^2+ ^ATPase; PML: promyelocytic leukemia protein; PP2a: protein phosphatase 2a; PSS-1: phosphatidylserine synthase-1; PTP: permeability transition pore; ROCs: receptor operated Ca^2+ ^channels; ROS: reactive oxygen species; SERCA: sarco-endoplasmic reticulum Ca^2+ ^ATPase; Sig-1R: Sigma-1 receptor; SMOCs: second messenger operated Ca^2+ ^channels; SPCA: secretory-pathway Ca^2+^-ATPase; SR: sarcoplasmic reticulum; UCP: uncoupling protein; VDAC: voltage-dependent anion channel; VOCs: voltage operated Ca^2+ ^channels.

## Competing interests

The authors declare that they have no competing interests.

## Authors' contributions

SP, JMS, CA, AB, MB, EDM, CG, SM, SM, FP, AR, JD, MRW and PP contributed to the preparation of the manuscript and approval of its final version.
